# (7*R*,8*S*,8a*S*)-8-Hydr­oxy-7-phenyl­perhydro­indolizin-3-one

**DOI:** 10.1107/S1600536809018455

**Published:** 2009-05-23

**Authors:** Ľubomír Švorc, Viktor Vrábel, Štefan Marchalín, Peter Šafář, Jozef Kožíšek

**Affiliations:** a Institute of Analytical Chemistry, Faculty of Chemical and Food Technology, Slovak Technical University, Radlinského 9, SK-81237 Bratislava, Slovak Republic; b Institute of Organic Chemistry, Catalysis and Petrochemistry, Faculty of Chemical and Food Technology, Slovak Technical University, Radlinského 9, SK-81237 Bratislava, Slovak Republic; c Institute of Physical Chemistry and Chemical Physics, Faculty of Chemical and Food Technology, Slovak Technical University, Radlinského 9, SK-81237 Bratislava, Slovak Republic

## Abstract

In the title compound, C_14_H_17_NO_2_, the six-membered ring of the indolizine system adopts a chair conformation. In the crystal, mol­ecules form chains parallel to the *b* axis *via* inter­molecular O—H⋯O hydrogen bonds. The absolute mol­ecular configuration was assigned from the synthesis.

## Related literature

For industrial uses of indolizines, see: Jaung & Jung (2003 ▶); Rotaru *et al.* (2005 ▶); Delattre *et al.* (2005 ▶); Kelin *et al.* (2001 ▶). For biological uses, see: Nash *et al.* (1988 ▶); Molyneux & James (1982 ▶); Harrell (1970 ▶); Ruprecht *et al.* (1989 ▶); Liu *et al.* (2007 ▶); Smith *et al.* (2007 ▶); Gupta *et al.* (2003 ▶); Rosseels *et al.* (1982 ▶); Oslund *et al.* (2008 ▶); Ostby *et al.* (2000 ▶). For synthesis of indolizines, see: Chuprakov & Gevorgyan (2007 ▶); Yan & Liu (2007 ▶). For the synthesis methods used, see: Šafář *et al.* (2009 ▶). For structures related to the title compound, see: Švorc *et al.* (2009 ▶). For comparison of mol­ecular parameters, see: Camus *et al.* (2003 ▶); Lokaj *et al.* (1999 ▶); Brown & Corbridge (1954 ▶); Pedersen (1967 ▶). For a general analysis of puckering, see: Cremer & Pople (1975 ▶).
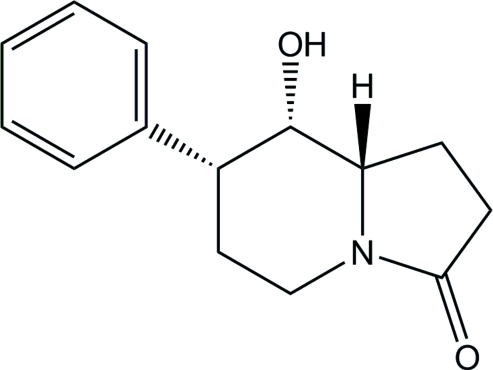


## Experimental

### 

#### Crystal data

C_14_H_17_NO_2_
*M* *_r_* = 231.29
Orthorhombic, 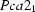

*a* = 11.4164 (3) Å
*b* = 6.6372 (2) Å
*c* = 15.5118 (4) Å
*V* = 1175.38 (6) Å^3^
*Z* = 4
Mo *K*α radiation
μ = 0.09 mm^−1^
*T* = 298 K
0.60 × 0.56 × 0.13 mm

#### Data collection

Oxford Diffraction Gemini R CCD diffractometer
Absorption correction: analytical (Clark & Reid, 1995 ▶) *T*_min_ = 0.901, *T*_max_ = 0.989
26298 measured reflections
1632 independent reflections
1128 reflections with *I* > 2σ(*I*)
*R*_int_ = 0.023

#### Refinement

*R*[*F* ^2^ > 2σ(*F* ^2^)] = 0.035
*wR*(*F* ^2^) = 0.101
*S* = 1.03
1632 reflections
157 parameters
1 restraint
H-atom parameters constrained
Δρ_max_ = 0.17 e Å^−3^
Δρ_min_ = −0.12 e Å^−3^

### 

Data collection: *CrysAlis CCD* (Oxford Diffraction, 2006 ▶); cell refinement: *CrysAlis RED* (Oxford Diffraction, 2006 ▶); data reduction: *CrysAlis RED*; program(s) used to solve structure: *SHELXS97* (Sheldrick, 2008 ▶); program(s) used to refine structure: *SHELXL97* (Sheldrick, 2008 ▶); molecular graphics: *DIAMOND* (Brandenburg, 2001 ▶); software used to prepare material for publication: *enCIFer* (Allen *et al.*, 2004 ▶).

## Supplementary Material

Crystal structure: contains datablocks I, global. DOI: 10.1107/S1600536809018455/bg2260sup1.cif

Structure factors: contains datablocks I. DOI: 10.1107/S1600536809018455/bg2260Isup2.hkl

Additional supplementary materials:  crystallographic information; 3D view; checkCIF report

## Figures and Tables

**Table 1:** Hydrogen-bond geometry (Å, °)

*D*—H⋯*A*	*D*—H	H⋯*A*	*D*⋯*A*	*D*—H⋯*A*
O2—H2⋯O1^i^	0.82	2.00	2.807 (2)	170

Symmetry code: (i) 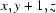.

**Fig. 1.**
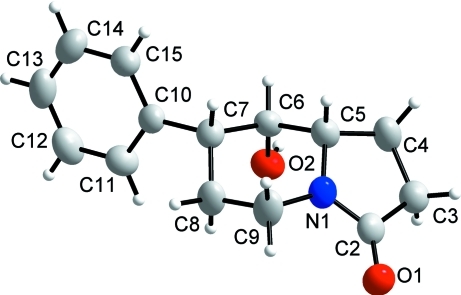
Molecular structure of (I) with the atomic numbering scheme. Displacement ellipsoids are drawn at the 50% probability level (Brandenburg, 2001).

**Fig. 2.**
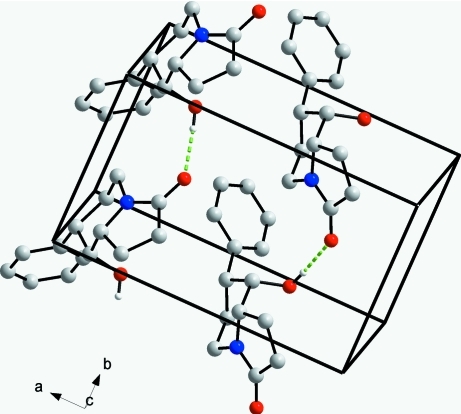
A packing of the molecule of (I), viewed along the b axis.

## supplementary crystallographic information

### Comment

Heterocycles are involved in a wide range of biologically important chemical reactions in living organisms, and therefore they form one of the most important and well investigated classes of organic compounds. One group of heterocycles, indolizines, has received much scientific attention during the recent years. They are known for their use as synthetic dyes (Jaung & Jung, 2003), fluorescent materials (Rotaru *et al.*, 2005; Delattre *et al.*, 2005) and also as key intermediates for the synthesis of indolizine based molecules (Kelin *et al.*, 2001). Indolizines both synthetic and natural have also been ascribed with a number of useful biological activities such as antibacterial, antiviral, antiinflammatory (Nash *et al.*, 1988; Molyneux & James, 1982), testosterone-3&-reductase inhibitors, 5-HT4 receptor antagonists, CNS depressants (Harrell *et al.*, 1970), anti-HIV (Ruprecht *et al.*, 1989), anti-cancer (Liu *et al.*, 2007; Smith *et al.*, 2007) and have been used for treating cardiovascular ailments (Gupta *et al.*, 2003). For instance, aminoalkyloxybenzenesulfonylindolizine compounds such as fantofarone and butoprozine have been used for the treatment of hypertension, arrhythmia and angina pectoris (Rosseels *et al.*, 1982). Several oxygenated indolizines have been shown to prevent, due to their strong anti-oxidative effects, the initiation of oxidation processes that lead to DNA damage (Oslund *et al.*, 2008; Ostby *et al.*, 2000). Consequently, synthesis of indolizines have attracted considerable attention and a number of synthetic methodologies have been developed for a variety of indolizines, making use of in particular, transition metal catalyzed reactions (Chuprakov & Gevorgyan, 2007; Yan & Liu, 2007). In addition, indolizines and their derivatives are important in the field of material science owing to their unique photophysical properties.

Based on these facts and in continutation of our interest in developing simple and efficient routes for the synthesis of novel indolizine derivatives, we report here the synthesis and molecular and crystal structure of the title compound, (I) (Fig. 1). A similar analysis of its enantiomer (the stereochemistry of atom C6 was confirmed as *R*) has already been published (Švorc *et al.*, 2009). The absolute configuration of (I) was established by the synthesis and is depicted in the scheme and Fig. 1. The expected stereochemistry of atoms C5, C6 and C7 was confirmed as S, S and *R*, respectively (Fig. 1). The central six-membered N-heterocyclic ring is not planar and adopts a chair conformation (Cremer & Pople, 1975). A calculation of least-squares planes shows that this ring is puckered in such a manner that the four atoms C5, C6, C8 and C9 are coplanar to within 0.010 (2) Å, while atoms N1 and C7 are displaced from this plane on opposite sides, with out-of-plane displacements of -0.555 (2) and 0.711 (2) Å, respectively. The phenyl ring attached to the indolizine ring system is planar (mean deviation is 0.009 (2) Å). The N1—C5 and N1—C9 bonds are approximately equivalent (See supplementary material) and both are much longer than the N1—C2 bond. Moreover, the N1 atom is *sp*^2^ hybridized, as evidenced by the sum of the valence angles around it [359.9 (2)°]. These data are consistent with conjugation of the lone-pair electrons on N1 with the adjacent carbonyl and agree with literature values for simple amides (Brown & Corbridge, 1954; Pedersen, 1967). The bond length of the carbonyl group C2═O1 is 1.236 (2) Å, respectively, is somewhat longer than typical carbonyl bonds. This may be due to the fact that atom O1 participates as acceptor in intermolecular hydrogen bonds with atom O2 as a donor. These intermolecular O—H···O hydrogen bonds link the molecules of (I) into extended chains, which run parallel to the *b* axis (Fig. 2) and help to stabilize the crystal structure of the compound. Bond lengths and angles in the indolizine ring system are in good agreement with values from the literature (Camus *et al.*, 2003; Lokaj *et al.*, 1999).

### Experimental

The title compound (7*R*,8S,8aS)-8-hydroxy-7-phenylhexahydroindolizin-3(5*H*)-one was prepared according literature procedures of Šafář *et al.* (2009).

### Refinement

All H atoms were placed in geometrically idealized positions and constrained to ride on their parent atoms, with C—H distances in the range 0.93–0.98 Å and O—H distance 0.85 Å and *U*_iso_ set at 1.2*U*_eq_ of the parent atom. The absolute configuration could not be reliably determined for this compound using Mo radiation, and has been assigned according to the synthesis; Friedel pairs have been merged.

### Crystal data

**Table:**

C_14_H_17_NO_2_	*F*(000) = 496
*M**_r_* = 231.29	*D*_x_ = 1.307 Mg m^−^^3^
Orthorhombic, *P**c**a*2_1_	Mo *K*α radiation, λ = 0.71073 Å
Hall symbol: P 2c -2ac	Cell parameters from 13180 reflections
*a* = 11.4164 (3) Å	θ = 3.3–29.4°
*b* = 6.6372 (2) Å	µ = 0.09 mm^−^^1^
*c* = 15.5118 (4) Å	*T* = 298 K
*V* = 1175.38 (6) Å^3^	Block, white
*Z* = 4	0.60 × 0.56 × 0.13 mm

### Data collection

**Table:**

Oxford Diffraction Gemini R CCD diffractometer	1632 independent reflections
Radiation source: fine-focus sealed tube	1128 reflections with *I* > 2σ(*I*)
graphite	*R*_int_ = 0.023
Detector resolution: 10.4340 pixels mm^-1^	θ_max_ = 29.4°, θ_min_ = 3.6°
Rotation method data acquisition using ω and φ scans	*h* = −15→15
Absorption correction: analytical (Clark & Reid, 1995)	*k* = −9→9
*T*_min_ = 0.901, *T*_max_ = 0.989	*l* = −20→21
26298 measured reflections	

### Refinement

**Table:**

Refinement on *F*^2^	Secondary atom site location: difference Fourier map
Least-squares matrix: full	Hydrogen site location: inferred from neighbouring sites
*R*[*F*^2^ > 2σ(*F*^2^)] = 0.035	H-atom parameters constrained
*wR*(*F*^2^) = 0.101	*w* = 1/[σ^2^(*F*_o_^2^) + (0.0644*P*)^2^ + 0.0334*P*] where *P* = (*F*_o_^2^ + 2*F*_c_^2^)/3
*S* = 1.03	(Δ/σ)_max_ < 0.001
1632 reflections	Δρ_max_ = 0.17 e Å^−^^3^
157 parameters	Δρ_min_ = −0.12 e Å^−^^3^
1 restraint	Extinction correction: *SHELXL97* (Sheldrick, 2008), Fc^*^=kFc[1+0.001xFc^2^λ^3^/sin(2θ)]^-1/4^
Primary atom site location: structure-invariant direct methods	Extinction coefficient: 0.014 (4)

### Special details

**Table:**

Experimental. face-indexed (*CrysAlis RED*; Oxford Diffraction, 2006)
Geometry. All e.s.d.'s (except the e.s.d. in the dihedral angle between two l.s. planes) are estimated using the full covariance matrix. The cell e.s.d.'s are taken into account individually in the estimation of e.s.d.'s in distances, angles and torsion angles; correlations between e.s.d.'s in cell parameters are only used when they are defined by crystal symmetry. An approximate (isotropic) treatment of cell e.s.d.'s is used for estimating e.s.d.'s involving l.s. planes.
Refinement. Refinement of *F*^2^ against ALL reflections. The weighted *R*-factor *wR* and goodness of fit *S* are based on *F*^2^, conventional *R*-factors *R* are based on *F*, with *F* set to zero for negative *F*^2^. The threshold expression of *F*^2^ > σ(*F*^2^) is used only for calculating *R*-factors(gt) *etc*. and is not relevant to the choice of reflections for refinement. *R*-factors based on *F*^2^ are statistically about twice as large as those based on *F*, and *R*- factors based on ALL data will be even larger.

### Fractional atomic coordinates and isotropic or equivalent isotropic displacement parameters (Å^2^)

**Table:**

	*x*	*y*	*z*	*U*_iso_*/*U*_eq_	
C2	0.2640 (2)	−0.3204 (3)	0.32882 (13)	0.0425 (5)	
C3	0.21718 (19)	−0.1773 (3)	0.26133 (17)	0.0490 (5)	
H3A	0.1398	−0.1296	0.2773	0.059*	
H3B	0.2121	−0.2438	0.2058	0.059*	
C4	0.3027 (2)	−0.0043 (4)	0.25750 (16)	0.0590 (6)	
H4A	0.2626	0.1227	0.2670	0.071*	
H4B	0.3409	0.0003	0.2017	0.071*	
C5	0.3932 (2)	−0.0428 (3)	0.32948 (15)	0.0466 (5)	
H5	0.4708	−0.0578	0.3033	0.056*	
C6	0.40010 (17)	0.1156 (3)	0.40092 (13)	0.0400 (4)	
H6	0.4340	0.2393	0.3771	0.048*	
C7	0.47970 (18)	0.0382 (3)	0.47386 (13)	0.0421 (5)	
H7	0.5558	0.0075	0.4478	0.051*	
C8	0.4309 (2)	−0.1608 (3)	0.50890 (16)	0.0514 (6)	
H8A	0.4814	−0.2100	0.5545	0.062*	
H8B	0.3537	−0.1378	0.5332	0.062*	
C9	0.4226 (2)	−0.3184 (3)	0.43821 (15)	0.0557 (6)	
H9A	0.5006	−0.3569	0.4197	0.067*	
H9B	0.3832	−0.4375	0.4600	0.067*	
C10	0.50152 (16)	0.1935 (3)	0.54318 (14)	0.0407 (5)	
C11	0.42606 (19)	0.2235 (4)	0.61193 (16)	0.0507 (5)	
H11	0.3582	0.1464	0.6159	0.061*	
C12	0.4494 (2)	0.3654 (4)	0.67466 (16)	0.0580 (6)	
H12	0.3976	0.3833	0.7203	0.070*	
C13	0.5498 (2)	0.4808 (4)	0.66972 (17)	0.0599 (7)	
H13	0.5662	0.5754	0.7122	0.072*	
C14	0.6248 (2)	0.4553 (4)	0.60216 (17)	0.0623 (7)	
H14	0.6919	0.5343	0.5982	0.075*	
C15	0.60156 (19)	0.3120 (4)	0.53928 (16)	0.0501 (5)	
H15	0.6538	0.2951	0.4938	0.060*	
N1	0.35736 (17)	−0.2359 (3)	0.36550 (12)	0.0473 (4)	
O1	0.22237 (14)	−0.4869 (2)	0.34724 (11)	0.0545 (5)	
O2	0.28771 (12)	0.1598 (2)	0.43433 (11)	0.0496 (4)	
H2	0.2647	0.2673	0.4146	0.074*	

### Atomic displacement parameters (Å^2^)

**Table:**

	*U*^11^	*U*^22^	*U*^33^	*U*^12^	*U*^13^	*U*^23^
C2	0.0503 (12)	0.0381 (10)	0.0391 (11)	0.0064 (9)	0.0004 (9)	−0.0066 (9)
C3	0.0507 (13)	0.0483 (11)	0.0480 (12)	0.0040 (9)	−0.0032 (10)	0.0014 (10)
C4	0.0858 (18)	0.0477 (12)	0.0436 (12)	−0.0088 (11)	−0.0148 (13)	0.0042 (10)
C5	0.0585 (13)	0.0404 (10)	0.0408 (10)	−0.0013 (9)	−0.0015 (10)	0.0023 (9)
C6	0.0488 (11)	0.0331 (10)	0.0382 (10)	−0.0015 (8)	0.0012 (9)	0.0038 (8)
C7	0.0395 (10)	0.0449 (12)	0.0419 (11)	0.0035 (8)	−0.0023 (9)	−0.0003 (9)
C8	0.0681 (15)	0.0384 (11)	0.0478 (11)	0.0032 (10)	−0.0163 (11)	0.0054 (9)
C9	0.0736 (15)	0.0366 (11)	0.0570 (14)	0.0078 (9)	−0.0211 (12)	0.0039 (10)
C10	0.0405 (10)	0.0417 (11)	0.0397 (10)	0.0017 (8)	−0.0059 (9)	0.0024 (8)
C11	0.0523 (11)	0.0496 (12)	0.0501 (12)	0.0022 (9)	0.0080 (11)	−0.0004 (10)
C12	0.0783 (16)	0.0509 (12)	0.0447 (12)	0.0132 (12)	0.0045 (12)	−0.0045 (11)
C13	0.0818 (17)	0.0480 (12)	0.0500 (13)	0.0058 (11)	−0.0209 (13)	−0.0055 (11)
C14	0.0621 (14)	0.0556 (14)	0.0693 (16)	−0.0098 (11)	−0.0195 (14)	0.0004 (12)
C15	0.0453 (11)	0.0587 (13)	0.0464 (12)	−0.0024 (10)	−0.0031 (10)	0.0035 (10)
N1	0.0593 (11)	0.0359 (9)	0.0465 (9)	0.0005 (8)	−0.0100 (8)	0.0014 (8)
O1	0.0640 (11)	0.0413 (8)	0.0582 (11)	−0.0047 (6)	−0.0065 (8)	0.0027 (7)
O2	0.0456 (8)	0.0486 (8)	0.0546 (9)	0.0083 (6)	0.0023 (7)	0.0085 (7)

### Geometric parameters (Å, °)

**Table:**

C2—O1	1.237 (2)	C8—C9	1.518 (3)
C2—N1	1.332 (3)	C8—H8A	0.9700
C2—C3	1.511 (3)	C8—H8B	0.9700
C3—C4	1.508 (3)	C9—N1	1.458 (3)
C3—H3A	0.9700	C9—H9A	0.9700
C3—H3B	0.9700	C9—H9B	0.9700
C4—C5	1.542 (3)	C10—C11	1.385 (3)
C4—H4A	0.9700	C10—C15	1.388 (3)
C4—H4B	0.9700	C11—C12	1.380 (3)
C5—N1	1.457 (3)	C11—H11	0.9300
C5—C6	1.530 (3)	C12—C13	1.380 (4)
C5—H5	0.9800	C12—H12	0.9300
C6—O2	1.414 (2)	C13—C14	1.364 (4)
C6—C7	1.540 (3)	C13—H13	0.9300
C6—H6	0.9800	C14—C15	1.388 (3)
C7—C10	1.510 (3)	C14—H14	0.9300
C7—C8	1.533 (3)	C15—H15	0.9300
C7—H7	0.9800	O2—H2	0.8200
			
O1—C2—N1	125.78 (19)	C9—C8—H8A	109.4
O1—C2—C3	125.9 (2)	C7—C8—H8A	109.4
N1—C2—C3	108.33 (17)	C9—C8—H8B	109.4
C2—C3—C4	106.09 (18)	C7—C8—H8B	109.4
C2—C3—H3A	110.5	H8A—C8—H8B	108.0
C4—C3—H3A	110.5	N1—C9—C8	109.40 (16)
C2—C3—H3B	110.5	N1—C9—H9A	109.8
C4—C3—H3B	110.5	C8—C9—H9A	109.8
H3A—C3—H3B	108.7	N1—C9—H9B	109.8
C3—C4—C5	106.18 (18)	C8—C9—H9B	109.8
C3—C4—H4A	110.5	H9A—C9—H9B	108.2
C5—C4—H4A	110.5	C11—C10—C15	117.7 (2)
C3—C4—H4B	110.5	C11—C10—C7	122.92 (19)
C5—C4—H4B	110.5	C15—C10—C7	119.4 (2)
H4A—C4—H4B	108.7	C10—C11—C12	121.4 (2)
N1—C5—C6	109.98 (17)	C10—C11—H11	119.3
N1—C5—C4	103.63 (18)	C12—C11—H11	119.3
C6—C5—C4	116.45 (19)	C13—C12—C11	120.0 (2)
N1—C5—H5	108.8	C13—C12—H12	120.0
C6—C5—H5	108.8	C11—C12—H12	120.0
C4—C5—H5	108.8	C14—C13—C12	119.7 (2)
O2—C6—C5	111.15 (17)	C14—C13—H13	120.2
O2—C6—C7	109.59 (16)	C12—C13—H13	120.2
C5—C6—C7	109.49 (16)	C13—C14—C15	120.3 (2)
O2—C6—H6	108.9	C13—C14—H14	119.9
C5—C6—H6	108.9	C15—C14—H14	119.9
C7—C6—H6	108.9	C10—C15—C14	121.0 (2)
C10—C7—C6	113.12 (15)	C10—C15—H15	119.5
C10—C7—C8	113.31 (18)	C14—C15—H15	119.5
C6—C7—C8	109.48 (17)	C2—N1—C5	115.52 (17)
C10—C7—H7	106.8	C2—N1—C9	125.54 (18)
C6—C7—H7	106.8	C5—N1—C9	118.92 (18)
C8—C7—H7	106.8	C6—O2—H2	109.5
C9—C8—C7	111.1 (2)		
			
O1—C2—C3—C4	−175.3 (2)	C8—C7—C10—C15	139.5 (2)
N1—C2—C3—C4	5.1 (2)	C15—C10—C11—C12	−0.3 (3)
C2—C3—C4—C5	−4.7 (2)	C7—C10—C11—C12	179.3 (2)
C3—C4—C5—N1	2.8 (2)	C10—C11—C12—C13	−0.1 (4)
C3—C4—C5—C6	−118.0 (2)	C11—C12—C13—C14	0.7 (4)
N1—C5—C6—O2	−67.5 (2)	C12—C13—C14—C15	−1.0 (4)
C4—C5—C6—O2	50.0 (2)	C11—C10—C15—C14	0.0 (3)
N1—C5—C6—C7	53.7 (2)	C7—C10—C15—C14	−179.6 (2)
C4—C5—C6—C7	171.18 (18)	C13—C14—C15—C10	0.6 (3)
O2—C6—C7—C10	−63.7 (2)	O1—C2—N1—C5	176.9 (2)
C5—C6—C7—C10	174.18 (17)	C3—C2—N1—C5	−3.5 (2)
O2—C6—C7—C8	63.7 (2)	O1—C2—N1—C9	−4.6 (3)
C5—C6—C7—C8	−58.4 (2)	C3—C2—N1—C9	175.0 (2)
C10—C7—C8—C9	−174.06 (18)	C6—C5—N1—C2	125.53 (19)
C6—C7—C8—C9	58.6 (2)	C4—C5—N1—C2	0.4 (2)
C7—C8—C9—N1	−52.9 (3)	C6—C5—N1—C9	−53.1 (3)
C6—C7—C10—C11	85.3 (2)	C4—C5—N1—C9	−178.2 (2)
C8—C7—C10—C11	−40.0 (3)	C8—C9—N1—C2	−126.4 (2)
C6—C7—C10—C15	−95.1 (2)	C8—C9—N1—C5	52.1 (3)

### Hydrogen-bond geometry (Å, °)

**Table:**

*D*—H···*A*	*D*—H	H···*A*	*D*···*A*	*D*—H···*A*
O2—H2···O1^i^	0.82	2.00	2.807 (2)	170

Symmetry codes: (i) *x*, *y*+1, *z*.
